# Aging increases UT‐B urea transporter protein abundance in brains of male mice

**DOI:** 10.14814/phy2.70175

**Published:** 2025-02-18

**Authors:** Farhana Pinki, Lauren McKeever, Derek A. Costello, Gavin Stewart

**Affiliations:** ^1^ UCD School of Biology and Environmental Science University College Dublin Dublin 4 Ireland; ^2^ UCD School of Biomolecular and Biomedical Science University College Dublin Dublin 4 Ireland; ^3^ UCD Conway Institute University College Dublin Dublin 4 Ireland

**Keywords:** aging, brain, protein, urea, UT‐B transporter

## Abstract

Facilitative UT‐B urea transporters in the brain play an important role in regulating levels of urea in various cell types, including astrocytes. Numerous studies have reported increased UT‐B RNA expression with aging and in neurological disorders, such as Alzheimer's Disease. However, much less is known about the effects of these conditions on UT‐B transporter protein abundance. This current study compared the levels of UT‐B RNA and protein in young and aged male C57BL/6 mice. Endpoint RT‐PCR experiments showed UT‐B RNA expression increased in both aged cortex and aged hippocampus. Importantly, these changes were coupled with an increase in protein abundance, as western blotting revealed that 30–35 kDa UT‐B1 protein was significantly increased in aged mouse brain tissues compared with tissue from young animals. An increased UT‐B1 protein abundance was observed in the hippocampus, cerebellum, frontal cortex, and occipital cortex. In contrast, no such changes were observed in the abundance of MCT1 short‐chain fatty acid transporters in these aged tissues. These data therefore confirmed that specific increases in UT‐B1 protein abundance occur in multiple regions of the aged male mouse brain. Further studies are now needed to determine cell‐specific changes and the functional consequence of increased UT‐B1 protein in aged brain tissues.

## 1 INTRODUCTION

Urea is the main nitrogenous breakdown product of protein catabolism in mammals, generated in the liver by the ornithine‐urea cycle. In the last decade, the classical view that a complete ornithine‐urea cycle is only present in the liver has been challenged. For example, detection of significant levels of urea in the brain, as well as key urea cycle enzymes, metabolites, and transporters, has revealed the presence of a functional urea cycle that caters towards neuronal homeostasis (Huang et al., 2021; Ju et al., 2022). It is now accepted that levels of urea in the brain increase in a variety of pathological conditions, such as Alzheimer's Disease (AD) (Xu et al., 2016), vascular dementia in the aging brain (Philbert et al., 2023), Parkinson's Disease (Scholefield et al., 2021), and a model for Huntington's disease (Handley et al., 2017). Recent research has shown the conversion of urea cycle metabolism occurs in astrocytes from mouse models of AD and, crucially, increased levels of certain urea cycle enzymes in the hippocampus of AD patients (Ju et al., 2022). Increased expression of urea and its nitrogenous precursor ammonia are toxic to the brain, as seen in uremic encephalopathy (Rosner et al., 2022), so understanding how the brain deals with these challenges is of vital importance. For example, the long‐term effects of silencing specific urea cycle enzymes in astrocytes from the AD brain are currently being investigated (Bhalla & Lee, 2024).

Another key element to broadening understanding of the physiological handling of urea in the brain is determining the precise mechanisms that regulate urea transport across cell membranes. Since the 1990s, it has been well established that mammalian facilitative urea transporters enable rapid movement of urea across plasma cell membranes and are encoded by either the SLC14A2 (UT‐A) gene (You et al., 1993) or the SLC14A1 (UT‐B) gene (Olives et al., 1994). With both genes, various isoforms are produced via alternative splicing, with six UT‐A isoforms (UT‐A1 to UT‐A6) (Smith & Fenton, 2006) and two UT‐B isoforms (UT‐B1 and UT‐B2) (Stewart, 2011) so far being characterized. Whilst UT‐A transporters main physiological role is as part of the renal urinary concentrating mechanism, UT‐B transporters have a variety of functions in tissues such as bladder, prostate, colon, and brain (Stewart, 2011).

In the context of the central nervous system (CNS), UT‐B transporters were originally reported in astrocytes from rat brain (Couriaud et al., 1996), but have subsequently been reported in other cell types, including neurons (Huang et al., 2021), and species, such as humans (Huang et al., 2021) and mice (Lucien et al., 2005). UT‐B transporters play a primary role in regulating urea concentration in the CNS (Yu et al., 2021), and studies of UT‐B knockout mice have previously revealed increased levels of urea in the hippocampus that is coupled with depression‐like behavior (Li et al., 2012). Previous studies have also linked changes in UT‐B expression to a variety of neurodegenerative diseases in humans (Recabarren & Alarcon, 2017; Wirz et al., 2013). Intriguingly, altered brain UT‐B transporter expression has also been reported to occur in other brain‐related medical conditions, including non‐suicidal self‐injury in adolescents (Guo et al., 2023) and chronic alcoholism (McClintick et al., 2013). Moreover, we have previously reported a potential role for UT‐B in the regulation of microglial activation under inflammatory conditions (Jones et al., 2021). Taken together, these studies point towards a role for UT‐B in the regulation of brain health and under disease conditions, highlighting the importance of better understanding its regulation.

Earlier studies have reported that expression of UT‐B RNA (Berger et al., 1998) and protein (Pinki et al., 2023) was prevalent throughout the rat brain. Furthermore, rat brain UT‐B protein abundance has been shown to increase with aging (Trinh‐Trang‐Tan et al., 2003) and in C6 astrocyte cells following exposure to urea (Pinki et al., 2023). However, little is known regarding UT‐B in the aging mouse brain, or whether there are region‐specific differences in its expression or abundance. It is also uncertain whether both UT‐B1 and UT‐B2 isoforms may play a role in the aging brain, as both isoforms have been reported in various other tissues such as bladder (Walpole et al., 2014) and colon (Walpole et al., 2018). The aim of this current study was therefore to investigate the impact of aging on the expression and abundance of UT‐B transporters in various regions of the mouse brain.

## 2 METHODS

### 2.1 Animals

Young (3–5 months old) and aged (18–24 months old) male C57BL/6 mice were euthanized by cervical dislocation and decapitation. Brains were isolated and submerged in ice‐cold phosphate‐buffered saline (PBS). Tissue was then rapidly dissected, flash frozen in liquid nitrogen, and stored at −80°C for later isolation of RNA and protein. All experimental animals were utilized following approval by the UCD Animals Ethics Committee, and brain tissue was kindly provided by Assoc. Prof. J. Baugh in accordance with a post‐mortem tissue‐sharing initiative. These experiments complied with ARRIVE guidelines and were in accordance with EU Directive 2010/63 for the protection of animals used for scientific purposes.

### 2.2 Rt‐PCR

Mouse brain cortex and hippocampus total RNA samples were extracted with an isolation protocol utilizing RNA‐STAT 60 (CS‐110, AMS Biotechnology, UK), BCP, isopropanol, and ethanol. The isolated RNA samples were treated with an Ambion TURBO DNA‐free kit (AM1907, Thermo Fisher Scientific, USA) with Turbo DNase enzyme for 25 min at 37°C, then quantified using a NanoDrop 1000 Spectrophotometer (Thermo Fisher Scientific, USA). Using these prepared mouse brain RNA samples, cDNA was prepared using the SensiFast cDNA synthesis kit (BIO‐65053, Bioline, UK). Next, using Go‐Taq polymerase (M5122, MyBio Ltd., Ireland), PCR amplification of the cDNA samples was performed with primers for GAPDH, PGK1, or UT‐B (both UT‐B1 and UT‐B2) in a Biometra T3000 thermocycler (Biometra, Germany). The cycling parameters for PCR reactions were as follows: 4 minutes initial denaturation at 94°C; 35 cycles of denaturation at 94°C for 30 s; annealing at 55°C or 60°C for 30 s; extension at 72°C for 30 s; a final extension at 72°C for 10 min. Identity of PCR products was confirmed using direct sequencing (Eurofins Genomics, Germany).

### 2.3 Antibodies

The previously characterized UT‐Bc19 antibodies (Walpole et al., 2014), raised against a 19‐amino acid peptide C‐terminal sequence of human UT‐B1 (NH2‐EENRIFYLQAKKRMVESPL‐COOH), were used to detect mouse UT‐B transporter proteins. Commercial antibodies raised against MCT1 short‐chain fatty acid transporters (AB1286‐I, Millipore, UK) were also used. These two primary antibodies were used in connection with horseradish peroxidase‐conjugated secondary anti‐rabbit IgG antibody (65–6120, Biosciences Ltd., UK) and anti‐chicken IgG antibody (A16054, Biosciences Ltd., UK), respectively.

### 2.4 Cell lines

C8D1A astrocyte cells were cultured in DMEM/F‐12 (Dulbecco's Modified Eagle Medium/Nutrient Mixture F‐12) (Lonza, USA) containing 20% heat‐inactivated FBS (fetal bovine serum) and 1% penicillin–streptomycin at 37°C with 5% CO_2_. For urea treatment experiments, C8D1A cells were incubated in 0–20 mM urea for a period of 24 h prior to RNA and protein preparation.

### 2.5 Western blotting

Mouse brain tissue samples were homogenized in homogenization buffer (300 mM mannitol, 12 mM HEPES, pH 7.6), using a Polytron PT1200 E homogenizer (Kinematica, Switzerland). Samples were centrifuged at 1000 ***g*** for 5 min at 4°C. The resulting supernatant was further centrifuged at 16,300 ***g*** for 30 min at 4°C, and the final pellet was suspended in homogenization buffer to produce membrane‐enriched protein samples. These protein samples were then mixed at a 1:3 ratio with 4X Laemmli buffer [1% SDS, 10% glycerol, 31.5 mM Tris–HCl (pH 6.8), and bromophenol blue] and heated at 70°C for 10 min before being loaded (~10–20 μg per lane) on gels. SDS‐PAGE gels composed of 12% resolving gel and 4% stacking gel were prepared in the laboratory and run in a vertical tank (Thermo Fisher Scientific, USA) or Criterion TGX precast 8%–16% protein gels (Bio‐Rad, USA) were used and run in Criterion cells (Bio‐Rad, USA). The proteins that were separated by size were next transferred to a nitrocellulose membrane. The resulting western blots were then incubated overnight at room temperature with either 1:1000 hUT‐Bc19 or 1:2000 anti‐MCT1 primary antibodies. [NOTE: For peptide inhibition experiments, hUT‐Bc19 was preincubated with specific or non‐specific immunizing peptide (1 μg peptide/1 μL hUTBc19 antibody) for 24 h using a rotating mixer.] Blots were washed and incubated with horseradish peroxidase‐conjugated anti‐rabbit antibody or anti‐chicken antibody (1:5000) for 1 h at room temperature. Finally, immunoreactive bands were detected using Western Lightning Plus ECL reagent (NEL104001EA, Perkin‐Elmer, USA) and a LAS‐4000 Imager (Fujifilm, Japan). Densitometry analysis of the resulting images was performed using ImageJ software (National Institute of Health, USA). For analysis of the UT‐B protein abundance, UT‐B signals were then normalized by comparison to the total amount of protein present, as obtained from an identically loaded Coomassie‐stained gel.

### 2.6 Statistical analysis

Data are shown as mean ± SEM (standard error mean), with *N* representing the number of samples. Unpaired *t*‐tests were performed, with groups deemed to be statistically significant when *p* < 0.05 (Instat, GraphPad, USA).

## 3 RESULTS

**FIGURE 1:**
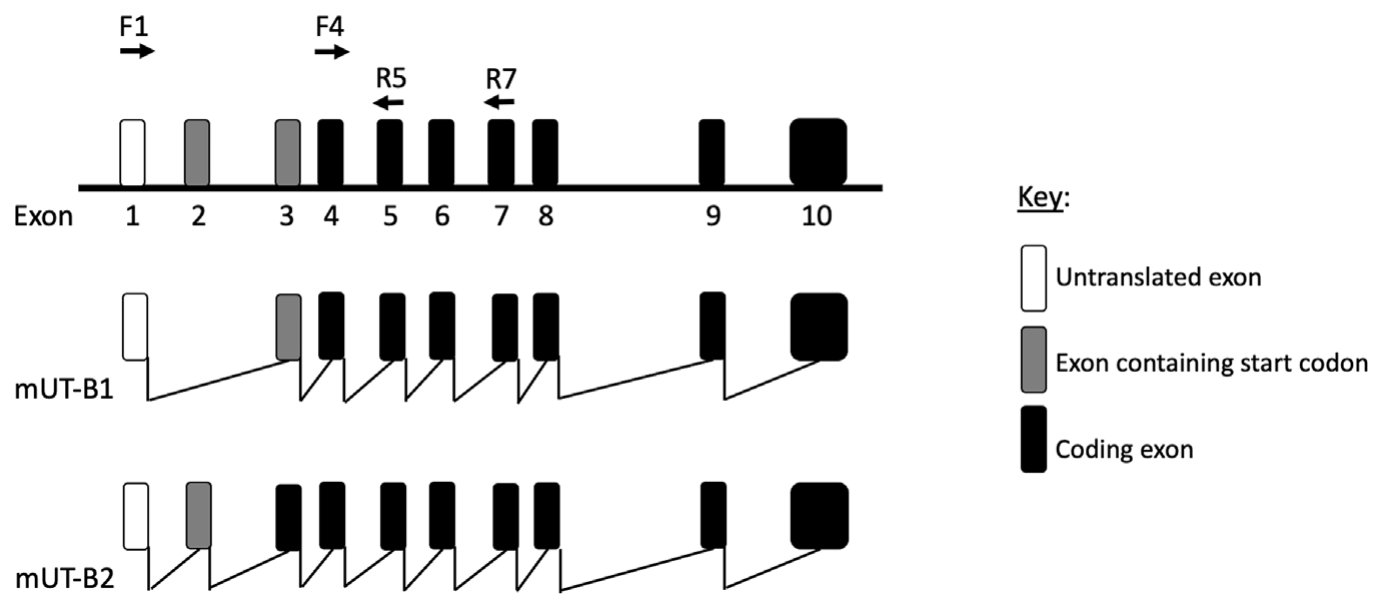
Schematic diagram of the mouse UT‐B gene structure. The UT‐B gene contains 10 exons, which are differentially transcribed to produce either UT‐B1 or UT‐B2 transcripts. The positions of various forward (F1 and F4) and reverse (R5 and R7) primers used for detecting UT‐B isoforms in PCR experiments are also shown.

**FIGURE 2:**
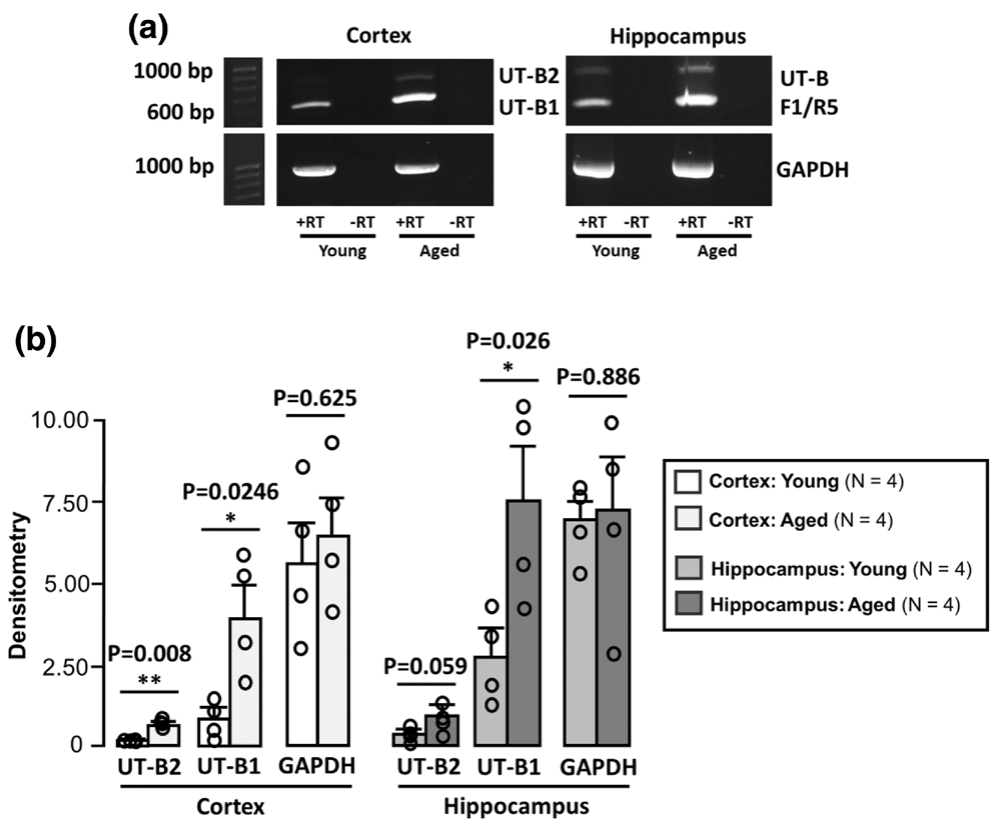
UT‐B1 and UT‐B2 RNA expression in young and aged mouse brain. (a) RT‐PCR experiments using F1/R5 UT‐B primer sets to detect both UT‐B1 and UT‐B2 RNA expression in young (3–5 months) and aged (18–24 months) mouse brain samples. +RT, +ve Reverse Transcriptase reaction; −RT, −ve Reverse Transcriptase reaction. (b) Bar graphs summarizing the densitometry data for mUT‐B1, mUT‐B2 and GAPDH expression in all cortex and hippocampus. * = *p* < 0.05, ** = *p* < 0.01.

UT‐B primers (Table 1) were designed against different exons in the mouse SLC14A1 gene, which encodes for two distinct UT‐B isoforms, mUT‐B1 and mUT‐B2 (Figure 1). RT‐PCR analysis carried out using the F1/R5 primer set identified that mUT‐B1 was strongly expressed in both cortex and hippocampus, whilst expression of mUT‐B2 was only weakly detected (Figure 2a). Densitometric analysis further revealed that expression of both mUT‐B1 (*p* < 0.05, *N* = 4, unpaired *t*‐test) and mUT‐B2 (*p* < 0.01, *N* = 4, unpaired *t*‐test) were significantly increased in aged cortex, compared to younger counterparts, whereas GAPDH expression was not (NS, *N* = 4, unpaired *t*‐test) (Figure 2b). A similar increase was observed for mUT‐B1 in the aged hippocampus (*p* < 0.05, *N* = 4, unpaired *t*‐test), although no statistically significant increases were detected for mUT‐B2 or GAPDH (NS, *N* = 4, unpaired *t*‐test) (Figure 2b).

**TABLE 1:** Summary of all primers used for investigations of UT‐B RNA expression in the mouse brain.

Primer	Sequence (5′ to 3′)
GAPDH_for	TGAAGGTCGGAGTCAACGGATTTGGT
GAPDH_rev	CATGTCGGCCATGAGGTCCACCAC
PGK1_for	GGTGGACTTCAACGTTCCTATG
PGK1_rev	CTAAACATTGCTGAGAGCATCC
UT‐B F1	CTTTATCTGGAGCCGCCTTCTGC
UT‐B F4	CCCTCTTGCTTAGCCAAGACAG
UT‐B R5	CAAGTCATAGACATAGCAGATACAGGG
UT‐B R7	GAGGAGAGCAGGATAGCACATAG

Abbreviations: For/F, forward; rev/R, reverse.

**FIGURE 3:**
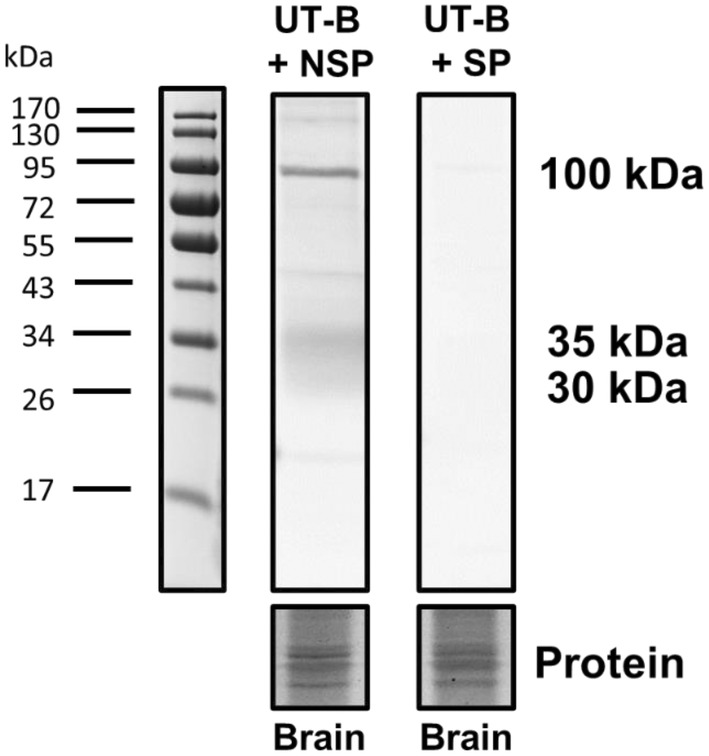
Peptide inhibition of UT‐Bc19 antibody signals detected in the mouse brain. Western blots of mouse brain protein samples (~10 μg per lane) probed with 1:1000 UT‐Bc19 antibody, pre‐incubated with either non‐specific (NSP) immunizing peptide (UT‐A6) or specific (SP) immunizing peptide (c19). Strong 30–35 kDa and 100 kDa signals were detected with the UT‐Bc19 antibody after NSP incubation. In contrast, these signals were almost completely absent after SP incubation. NSP, Non‐specific peptide; SP, Specific peptide.

UT‐Bc19 antibodies (hUT‐Bc19) have previously been validated for detection of UT‐B protein in human tissues (Walpole et al., 2014). In the current study, we utilized hUT‐Bc19 to investigate the abundance of UT‐B protein in samples from young and aged mouse brain tissues. Peptide inhibition experiments were performed with UT‐Bc19 antibodies using whole mouse brain protein samples (Figure 3). Importantly, signals corresponding to the 30–35 kDa UT‐B1 protein, detected after pre‐incubation in non‐specific peptide (UT‐A6), were not visible when the antibody was pre‐incubated with specific peptide (c19 peptide). A strong immunoreactive band observed at 100 kDa was also not detected following peptide inhibition (Figure 3), confirming its specificity for detection by the hUT‐Bc19 antibodies.

**FIGURE 4:**
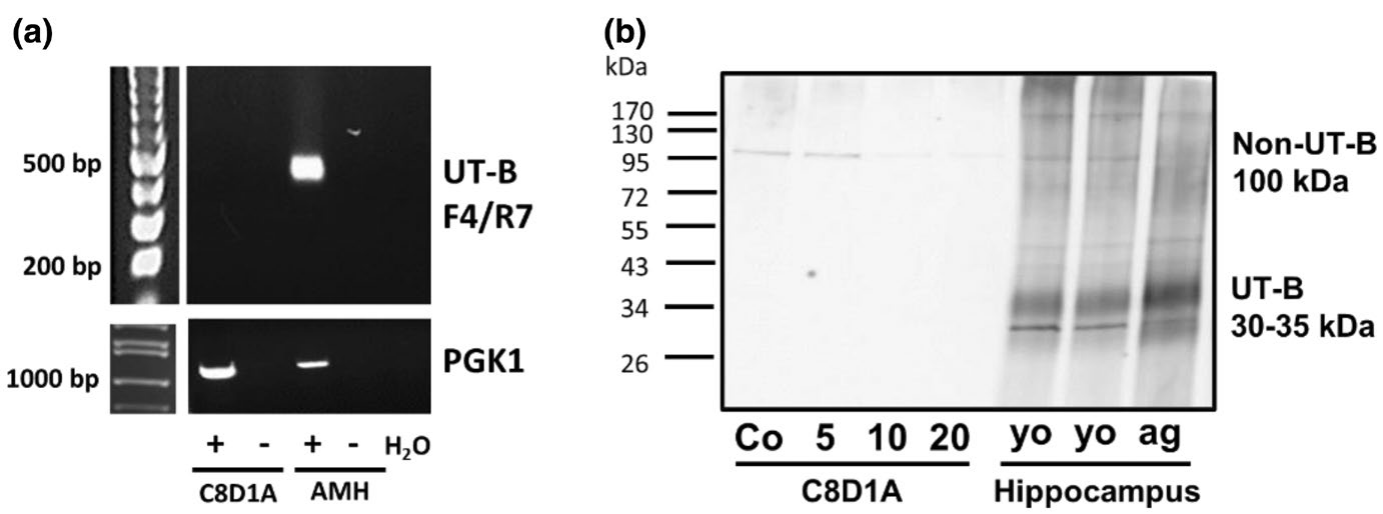
UT‐B RNA expression and protein abundance in C8D1A mouse astrocyte cells, and hippocampal tissue from young and aged mice. (a) RT‐PCR experiments using C8D1A astrocyte cell and aged mouse hippocampus cDNA samples. Using F4/R7 primers, no UT‐B expression was detected in C8D1A cells, whereas a strong PGK1 signal was detected. (b) Western blots of urea‐treated C8D1A, young (3–5 months) hippocampus and aged (18–24 months) hippocampus protein samples (~10 μg per lane) probed with 1:1000 UT‐Bc19 antibodies. The strong 30–35 kDa UT‐B signals detected in all hippocampus samples were absent in the C8D1A protein samples. In contrast, the non‐UT‐B 100 kDa signal is present in C8D1A protein and was decreased with urea treatment. +, +ve Reverse Transcriptase reaction; −, −ve Reverse Transcriptase reaction; AMH, Aged Mouse Hippocampus; Co, Control; 5, 5 mM urea (24 h); 10, 10 mM urea (24 h); 20, 20 mM urea (24 h); ag, aged; yo, young.

Following previously reports of UT‐B expression in rat astrocyte cells (Couriaud et al., 1996) and the rat C6 astrocyte cell line (Pinki et al., 2023), we carried out investigation to determine its abundance in the mouse C8D1A astrocyte cell line. However, these experiments revealed that these cells did not express detectable levels of UT‐B RNA (Figure 4a), which was coupled with an absence of UT‐B1 protein (30–35 kDa) (Figure 4b). In contrast, UT‐B1 protein was detected in both young and aged mouse hippocampus (Figure 4b). The 100 kDa protein signal was detected in C8D1A cells, similarly to that detected in hippocampal tissue. However, this appeared to be ablated in cells exposed to extracellular urea in a concentration‐dependent manner (Figure 4b). These findings suggest that the 100 kDa band detected by the hUT‐Bc19 antibody did not correspond to UT‐B and should be classified as a non‐UT‐B protein signal.

**FIGURE 5:**
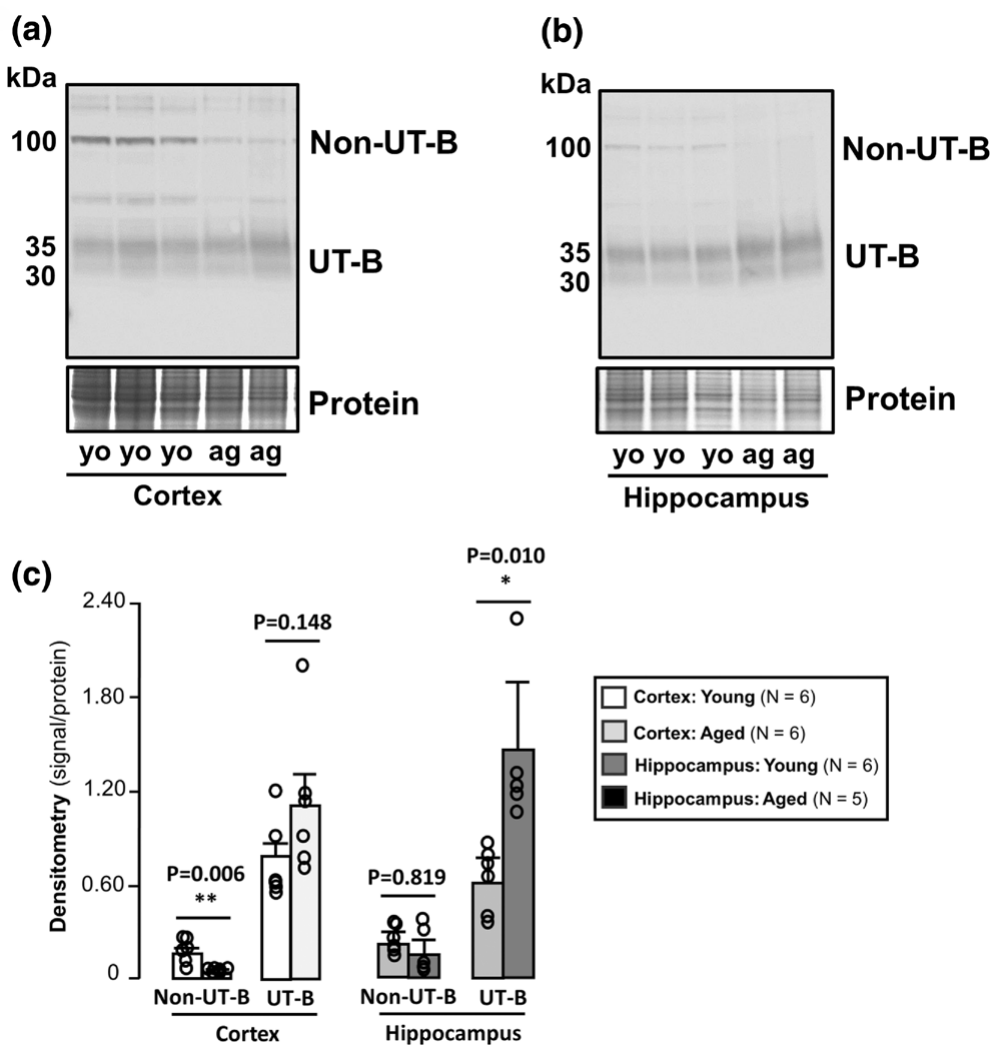
UT‐B protein abundance in cortex and hippocampus from young and aged mice. (a) Western blot of young (3–5 months) and aged (18–24 months) mouse cortex protein samples (~10 μg per lane) probed with 1:1000 UT‐Bc19 antibodies. Strong 30–35 kDa UT‐B signals were similar between young and aged samples. In contrast, non‐UT‐B 100 kDa signals were much reduced in aged samples. (b) Western blot of young (3–5 months) and aged (18–24 months) mouse hippocampus protein samples (~10 μg per lane) probed with 1:1000 UT‐Bc19 antibodies. Strong 30–35 kDa UT‐B signals were increased in aged hippocampus samples, whereas non‐UT‐B 100 kDa signals were not increased. (c) Bar graphs summarizing densitometry data (normalization using UT‐B signal/protein) values for Non‐UT‐B and UT‐B protein abundance. ag, aged; Yo, young. * = *p* < 0.05, ** = *p* < 0.01.

To further explore age‐related and region‐specific changes in UT‐B1 protein abundance in the mouse brain, we carried out western blotting experiments using samples from cortex (Figure 5a) and hippocampus (Figure 5b) of young and aged mice. The UT‐Bc19 antibody detected strong immunoreactive bands corresponding to 30–35 kDa and 100 kDa in both cortex (Figure 5a) and hippocampus (Figure 5b) of young animals. There was no significant difference in the 30–35 kDa UT‐B1 signal detected within the cortex of aged compared with young mice (*N* = 6, unpaired *t*‐test) (Figure 5c). However, the 100 kDa non‐UT‐B signal was significantly reduced in the aged cortex relative to that seen in equivalent samples from young animals (*p* < 0.01, *N* = 6, Unpaired *t*‐Test) (Figure 5c). In contrast, there was a significant increase in 30–35 kDa UT‐B1 protein abundance in the aged hippocampus compared with samples from young mice (*p* < 0.05, *N* = 5, unpaired *t*‐test). While the 100 kDa signal appeared reduced in the aged hippocampus, this was not significantly different to that detected in young animals (*N* = 5, unpaired *t*‐test) (Figure 5c).

**FIGURE 6:**
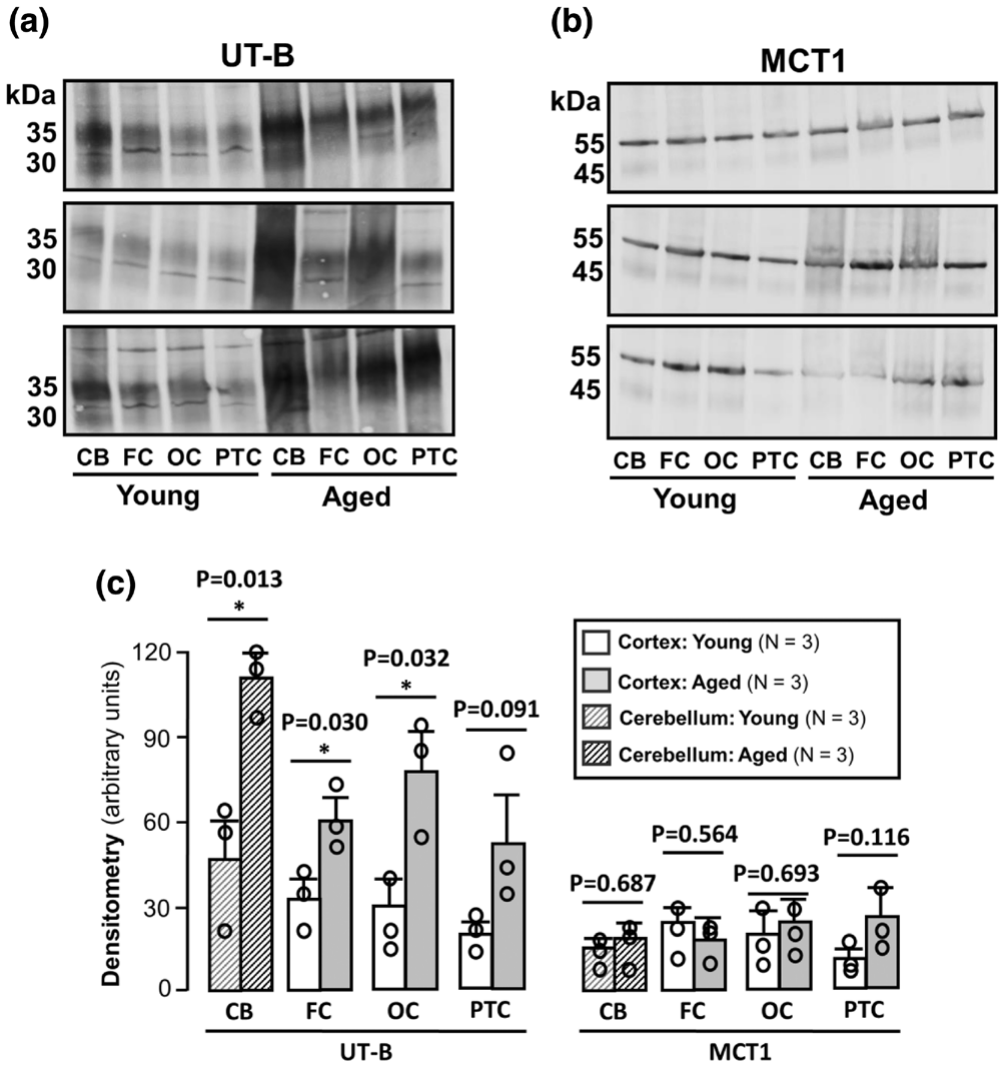
UT‐B and MCT1 protein abundance in specific brain regions from young and aged mice. Western blots of cerebellum, frontal cortex, occipital cortex, and parietal/temporal cortex protein samples (~10 μg per lane) probed with (a) 1:1000 UT‐Bc19 and (b) 1:2000 MCT1 antibodies. The abundance of the 30–35 kDa UT‐B protein was increased in all aged samples, whereas no such changes were observed for MCT1 protein. (c) Bar graphs summarizing densitometry values for UT‐B and MCT1 abundance across young and aged mouse cerebellum, hippocampus, frontal cortex, occipital cortex, and parietal/temporal cortex protein samples. CB, Cerebellum; FC, Frontal Cortex; OC, Occipital Cortex; PTC, Parietal/Temporal Cortex. * = *p* < 0.05.

Having revealed age‐related changes in UT‐B1 abundance in hippocampus, which were not consistently identified in general cortex samples, we next sought to further examine region‐specific changes in the mouse brain. UT‐B1 protein was reliably detected in all assessed samples from cerebellum, frontal cortex, occipital cortex, and parietal/temporal cortex isolated from both young and aged mice (Figure 6a). Similar to that seen in hippocampus, an age‐related increase in UT‐B1 protein abundance was observed in cerebellum, frontal cortex and occipital cortex (*p* < 0.05, *N* = 3, unpaired *t*‐test). However, although there was a trend observed for increases in aged samples from parietal/temporal cortex as well, this was not a significant change (*N* = 3, unpaired *t*‐test) (Figure 6c). To determine whether the age‐associated changes were specific to UT‐B1, abundance of the monocarboxylate transporter, MCT1, was used as a comparator. While MCT1 was similarly detected across all brain regions, no significant changes were observed between aged and young animals (*N* = 3, unpaired *t*‐test) (Figure 6b,c).

## 4 DISCUSSION

Endpoint RT‐PCR experiments revealed moderate expression of mUT‐B1 and weak expression of mUT‐B2 in young male mouse brain samples (Figure 2a). This pattern is the same that has been reported in all other tissues, except for rumen where UT‐B2 is the predominant transcript (Stewart et al., 2005). In aged mouse brain, both mUT‐B1 and mUT‐B2 expression were significantly increased in the cortex samples, whereas only mUT‐B1 was significantly increased in hippocampal samples (Figure 2b). This data confirms observations in UT‐B knockout mice that the hippocampus is an important site regarding urea transport (Li et al., 2012), but also indicates cortical regions may also be crucial.

To confirm whether these changes were also occurring in UT‐B protein abundance, western blotting experiments were performed. Initial experiments using whole male mouse brain samples detected two main signals at 30–35 kDa and at 100 kDa (Figure 3). As these signals were all prevented by pre‐incubation of the hUTBc19 with its specific immunizing peptide, it can be stated that they are all due to the hUTBc19 antibodies and not antibody contamination. The 30–35 kDa signals are very likely to be UT‐B1 proteins given: (i) they are identical to those detected in our previous rat brain studies with these antibodies (Pinki et al., 2023); (ii) they are similar to the non‐glycosylated 29 kDa and N‐glycosylated 32.5 kDa UT‐B1 proteins in rat brain reported by another group 20 years ago using different polyclonal antibodies also raised to the C‐terminal of UT‐B1 (Trinh‐Trang‐Tan et al., 2003); (iii) the size for UT‐B2 proteins previously detected by the hUTBc19 antibodies in deer rumen was ~50 kDa (Zhong et al., 2022). However, our previous studies with rat brain also showed that a similar 100 kDa signal was not actually a UT‐B protein (Pinki et al., 2023). Next, experiments using the mouse astrocyte C8D1A cell line further confirmed the nature of these signals. Despite these cells not expressing UT‐B RNA (Figure 4a), the 100 kDa signal was again detected with hUTBc19 and was ablated with 24‐hour treatment in 10 mM or 20 mM urea (Figure 4b). In contrast, the 30–35 kDa UT‐B1 signals were not detected in C8D1A cells but were present in hippocampus samples (Figure 4b). UT‐B has previously been reported in astrocytes of rat origin (Berger et al., 1998; Pinki et al., 2023). Moreover, its transport function in astrocytes is regulated by osmolarity and urea concentration (Ogami et al., 2006). It is somewhat surprising, therefore, that UT‐B was not identified in mouse C8D1A cells in the current study. However, enzymes related to the urea cycle are preferentially expressed in the AD brain (Ju et al., 2022), suggestive of enhanced functioning of UT‐B under disease conditions. We might speculate, therefore, that under conditions of inflammatory stress or challenge that mimic the astrogliosis associated with disease, UT‐B expression and abundance might be upregulated in C8D1A cells. Indeed, this is further supported by our findings in tissue from male‐aged mice.

Comparisons of protein abundance in young and aged mice revealed some interesting changes. In aged general samples of cortex, there was no change in the 30–35 kDa UT‐B1 signal, though there was a significant decrease in the 100 kDa non‐UT‐B signal (Figure 5a). Given that it is known that urea levels in the brain generally increase with aging, this latter finding agrees with the data from Figure 4 showing that increased urea levels may ablate this non‐UT‐B protein. In the aged hippocampus, there was a significant increase in 30–35 kDa UT‐B1 protein and no change in the 100 kDa protein (Figure 5b). These data for UT‐B1 protein abundance in the hippocampus in the aged brain therefore agree with the RNA expression data.

Finally, given the variability of total cortex data, more specific regions of mouse brain were investigated. Like our previous observation in rats (Pinki et al., 2023), UT‐B1 appeared to be relatively equally distributed throughout individual regions of the mouse brain. Interestingly, significant increases in 30–35 kDa UT‐B1 proteins were observed in aged samples from cerebellum, frontal cortex, and occipital cortex (Figure 6a). To ensure that these differences were not due to variation in the quality of protein samples obtained from the young and aged samples, antibodies raised against the MCT1 short chain fatty acid transporter were also utilized. The expected 45 and 55 kDa MCT1 signals (Al‐mousawi et al., 2016) were detected in all samples and, crucially, no significant differences were observed between any of the young and aged samples (Figure 6b). This adds weight to the interpretation that observed increases in UT‐B1 protein abundance in aged mouse brain were physiologically relevant and therefore in agreement with a previous study in aged rats (Trinh‐Trang‐Tan et al., 2003).

In conclusion, this study has confirmed that aging causes an increase in the expression of both mUT‐B1 and mUT‐B2 in the male mouse brain. It has shown that aging specifically increases the abundance of UT‐B1 transporter protein in the hippocampus, cerebellum, frontal cortex, and occipital cortex regions. These findings agree with recent reports into the changes in brain urea metabolism that occur with aging and neurodegenerative diseases. Future studies should investigate whether the exact same cellular changes occur within aged female mouse brain tissues. Crucially, there should also be a focus on the functional consequences of these changes and investigate whether UT‐B1 proteins are a potential target for clinical interventions.

## FUNDING INFORMATION

The authors would like to thank the Jack Pickard Research Foundation for funding that provided consumables for the project. The Foundation played no role in the design of the study, collection, analysis, and interpretation of data; in the writing of the report, or in the decision to submit the article for publication.

## CONFLICT OF INTEREST STATEMENT

No conflicts of interest, financial, or otherwise, are declared by the author(s).

## References

[phy270175-bib-0001] Al‐mousawi, H. , Ryan, E. , McGrane, A. , Riveros‐Beltran, S. , Walpole, C. , Dempsey, E. , Courtney, D. , Fearon, N. , Winter, D. C. , Baird, A. W. , & Stewart, G. (2016). Differential protein abundance of a basolateral MCT1 transporter in the human gastrointestinal tract. Cell Biology International, 40, 1303–1312. 27634412 10.1002/cbin.10684

[phy270175-bib-0002] Berger, U. V. , Tsukaguchi, H. , & Hediger, M. A. (1998). Distribution of mRNA for the facilitated urea transporter UT3 in the rat nervous system. Anatomy and Embryology, 197, 405–414. 9623675 10.1007/s004290050152

[phy270175-bib-0003] Bhalla, M. , & Lee, C. J. (2024). Long‐term inhibition of ODC1 in APP/PS1 mice rescues amyloid pathology and switches astrocytes from a reactive to active state. Molecular Brain, 17, 3. 38216963 10.1186/s13041-024-01076-8 PMC10785549

[phy270175-bib-0004] Couriaud, C. , Ripoche, P. , & Rousselet, G. (1996). Cloning and functional characterization of a rat urea transporter: Expression in the brain. Biochimica et Biophysica Acta, 1309, 197–199. 8982255 10.1016/s0167-4781(96)00172-8

[phy270175-bib-0005] Guo, Z. , Liu, Y. , Wang, C. , Li, S. , Yu, L. , Wu, W. , You, X. , Zhang, Y. , & Teng, Z. (2023). Exploring the association of addiction‐related genetic factors with non‐suicidal self‐injury in adolescents. Frontiers in Psychiatry, 14, 1126615. 37065902 10.3389/fpsyt.2023.1126615 PMC10102595

[phy270175-bib-0006] Handley, R. R. , Reid, S. J. , Brauning, R. , Maclean, P. , Mears, E. R. , & Fourie, I. (2017). Brain urea increase is an early Huntington's disease pathogenic event observed in a prodromal transgenic sheep model and HD cases. Proceedings of the National Academy of Sciences, 114, 1–10. 10.1073/pnas.1711243115 PMC5748180 29229845

[phy270175-bib-0007] Huang, B. , Wang, H. , Zhong, D. , Meng, J. , Li, M. , Yang, B. , & Ran, J. (2021). Expression of urea transporter B in Normal and injured brain. Frontiers in Neuroanatomy, 15, 1–11. 10.3389/fnana.2021.591726 PMC8194276 34122018

[phy270175-bib-0008] Jones, A. , Pinki, F. , Stewart, G. S. , & Costello, D. A. (2021). Inhibition of urea transporter (UT)‐B modulates LPS‐induced inflammatory responses in BV2 microglia and N2a neuroblastoma cells. Neurochemical Research, 46, 1322–1329. 33675462 10.1007/s11064-021-03283-4

[phy270175-bib-0009] Ju, Y. H. , Bhalla, M. , Hyeon, S. J. , Oh, J. E. , Yoo, S. , Chae, U. , Kwon, J. , Koh, W. , Lim, J. , Park, Y. M. , Lee, J. , Cho, I. J. , Lee, H. , Ryu, H. , & Lee, C. J. (2022). Astrocytic urea cycle detoxifies Aβ‐derived ammonia while impairing memory in Alzheimer's disease. Cell Metabolism, 34, 1104–1120. 35738259 10.1016/j.cmet.2022.05.011

[phy270175-bib-0010] Li, X. , Ran, J. , Zhou, H. , Lei, T. , Zhou, L. , Han, J. , & Yang, B. (2012). Mice lacking urea transporter UT‐B display depression‐like behavior. Journal of Molecular Neuroscience, 46, 362–372. 21750947 10.1007/s12031-011-9594-3

[phy270175-bib-0011] Lucien, N. , Bruneval, P. , Lasbennes, F. , Belair, M. F. , Mandet, C. , Cartron, J. P. , Bailly, P. , & Trinh‐Trang‐Tan, M. M. (2005). UT‐B1 urea transporter is expressed along the urinary and gastrointestinal tracts of the mouse. American Journal of Physiology Regulatory Physiology, 288, R1046–R1056. 10.1152/ajpregu.00286.2004 15563580

[phy270175-bib-0012] McClintick, J. , Xuei, X. , Tischfield, J. A. , Goate, A. , Foroud, T. , Wetherill, L. , Ehringer, M. A. , & Edenberg, H. J. (2013). Stress‐response pathways are altered in the hippocampus of chronic alcoholics. Alcohol, 47, 505–515. 23981442 10.1016/j.alcohol.2013.07.002 PMC3836826

[phy270175-bib-0013] Ogami, A. , Miyazaki, H. , Niisato, N. , Sugimoto, T. , & Marunaka, Y. (2006). UT‐B1 urea transporter plays a noble role as active water transporter in C6 glial cells. Biochemical and Biophysical Research Communications, 353, 619–624. 10.1016/j.bbrc.2006.10.097 17081500

[phy270175-bib-0014] Olives, B. , Neau, P. , Bailly, P. , Hediger, M. A. , Rousselet, G. , Cartron, J. P. , & Ripoche, P. (1994). Cloning and functional expression of a urea transporter from human bone marrow cells. Journal of Biological Chemistry, 269, 31649–31652. 7989337

[phy270175-bib-0015] Philbert, S. A. , Xu, J. , Scholefield, M. , Patassini, S. , Church, S. J. , Unwin, R. D. , Roncaroli, F. , & Cooper, G. J. S. (2023). Extensive multiregional urea elevations in a case‐control study of vascular dementia point toward a novel shared mechanism of disease amongst age‐related dementias. Frontiers in Molecular Neuroscience, 16, 1215637. 37520429 10.3389/fnmol.2023.1215637 PMC10372345

[phy270175-bib-0016] Pinki, P. , Costello, D. A. , & Stewart, G. (2023). Regional investigation of UT‐B urea transporters in the rat brain. Biochemistry and Biophysics Reports, 36, 101563. 37929290 10.1016/j.bbrep.2023.101563 PMC10624589

[phy270175-bib-0017] Recabarren, D. , & Alarcon, M. (2017). Gene networks in neurodegenerative disorders. Life Sciences, 183, 83–97. 28623007 10.1016/j.lfs.2017.06.009

[phy270175-bib-0018] Rosner, M. H. , Husain‐Syed, F. , Reis, T. , Ronco, C. , & Vanholder, R. (2022). Uremic encephalopathy. Kidney International, 101, 227–241. 34736971 10.1016/j.kint.2021.09.025

[phy270175-bib-0019] Scholefield, M. , Church, S. J. , Xu, J. , Patassini, S. , Roncaroli, F. , Hooper, N. M. , Unwin, R. D. , & Cooper, G. J. S. (2021). Severe and regionally widespread increases in tissue urea in the human brain represent a novel finding of pathogenic potential in Parkinson's disease dementia. Frontiers in Molecular Neuroscience, 14, 711396. 34751215 10.3389/fnmol.2021.711396 PMC8571017

[phy270175-bib-0020] Smith, C. P. , & Fenton, R. A. (2006). Genomic organization of the mammalian Slc14a2 urea transporter genes. Journal of Membrane Biology, 212, 109–117. 17264986 10.1007/s00232-006-0870-z

[phy270175-bib-0021] Stewart, G. (2011). The emerging physiological roles of the SLC14A family of urea transporters. British Journal of Pharmacology, 164, 1780–1792. 21449978 10.1111/j.1476-5381.2011.01377.x PMC3246703

[phy270175-bib-0022] Stewart, G. S. , Graham, C. , Cattell, S. , Smith, T. P. L. , Simmons, N. L. , & Smith, C. P. (2005). UT‐B is expressed in bovine rumen: Potential role in ruminal urea transport. American Journal of Physiology Regulatory Physiology, 289, R605–R612. 10.1152/ajpregu.00127.2005 15845882

[phy270175-bib-0023] Trinh‐Trang‐Tan, M. M. , Geelen, G. , Teillet, L. , & Corman, B. (2003). Urea transporter expression in aging kidney and brain during dehydration. American Journal of Physiology Renal Physiology, 283, F912–F922. 10.1152/ajpregu.00207.2003 12933359

[phy270175-bib-0024] Walpole, C. , Farrell, A. , McGrane, A. , & Stewart, G. S. (2014). Expression and localization of a UT‐B urea transporter in the human bladder. American Journal of Physiology. Renal Physiology, 307, F1088–F1094. 25209859 10.1152/ajprenal.00284.2014

[phy270175-bib-0025] Walpole, C. , McGrane, A. , Al‐mousawi, H. , Winter, D. , Baird, A. , & Stewart, G. S. (2018). Investigation of facilitative urea transporters in the human gastrointestinal tract. Physiological Reports, 6, e13826. 30101448 10.14814/phy2.13826 PMC6087735

[phy270175-bib-0026] Wirz, K. , Bossers, K. , Stargardt, A. , Kamphuis, W. , Swaab, D. , Hol, E. , & Verhaagen, J. (2013). Cortical beta amyloid protein triggers an immune response, but no synaptic changes in the APPswe/PS1dE9 Alzheimer's disease mouse model. Neurobiology of Aging, 34, 1328–1342. 23245294 10.1016/j.neurobiolaging.2012.11.008

[phy270175-bib-0027] Xu, J. , Begley, P. , Church, S. J. , Patassini, S. , Hollywood, K. A. , Jüllig, M. , Curtis, M. A. , Waldvogel, H. J. , Faull, R. L. M. , Unwin, R. D. , & Cooper, G. J. S. (2016). Graded perturbations of metabolism in multiple regions of human brain in Alzheimer's disease: Snapshot of a pervasive metabolic disorder. Biochimica et Biophysica Acta, 1862, 1084–1092. 26957286 10.1016/j.bbadis.2016.03.001 PMC4856736

[phy270175-bib-0028] You, G. , Smith, C. P. , Kanai, Y. , Lee, W. S. , Stelzner, M. , & Hediger, M. A. (1993). Cloning and characterization of the vasopressin‐regulated urea transporter. Nature, 365, 844–847. 8413669 10.1038/365844a0

[phy270175-bib-0029] Yu, L. , Liu, T. , Fu, S. , Li, L. , Meng, X. , Su, X. , Xie, Z. , Ren, J. , Meng, Y. , Lv, X. , & Du, Y. (2021). Physiological functions of urea transporter B. Pflügers Archiv – European Journal of Physiology, 471, 1359–1368. 10.1007/s00424-019-02323-x PMC6882768 31734718

[phy270175-bib-0030] Zhong, C. , Griffin, L. L. , Heussaff, O. , O'Dea, R. , Whelan, C. , & Stewart, G. (2022). Sex‐related differences in UT‐B urea transporter abundance in fallow deer rumen. Veterinary Sciences, 9, 73. 35202326 10.3390/vetsci9020073 PMC8878845

